# A cross sectional observational study to estimate herd level risk factors for *Leptospira* spp. serovars in small holder dairy cattle farms in southern Chile

**DOI:** 10.1186/1746-6148-10-126

**Published:** 2014-06-06

**Authors:** Miguel Salgado, Barbara Otto, Errol Sandoval, German Reinhardt, Sofia Boqvist

**Affiliations:** 1 Department of Biochemistry and Microbiology, Faculty of Sciences, Universidad Austral de Chile, Edificio Instapanel, Campus Isla Teja, CC 567 Valdivia, Chile; 2 Department of Biomedical Sciences and Veterinary Public Health, Swedish University of Agricultural Sciences, Box 7028, SE-750 07 Uppsala, Sweden

**Keywords:** *Leptospira*, Seropositivity, MAT, Iinfection, Risk factors, Herd

## Abstract

**Background:**

The south of Chile constitutes the main cattle milk producing area of the country. Regarding leptospirosis control in Chile, there is neither an official program nor an epidemiological characterization of smallholder dairy farms. This study was carried out to determine *Leptospira* seroprevalence and to evaluate risk factors associated with seropositivity at herd level in smallholder bovine dairy herds in southern Chile.

A cross-sectional study was conducted, and a convenient sample of 1,537 apparently healthy dairy cows was included in the study. Individual blood samples were taken and examined for six selected reference *Leptospira* serovars by the Microscopic Agglutination Test (MAT).

**Results:**

Of the included herds 75% (52/69) showed serological titers against one or more *Leptospira* serovar. *Leptospira borgpetersenii* serovar Hardjo was the serovar most frequently (81%) reported from animals with positive results. The variables considered risk factors for *Leptospira* seropositivity were calve natural breeding system, using a specific calving area and vaccination against *Leptospira*. Adult cows in contact with calves weaned, proved to be a protective factor against infection.

**Conclusions:**

Herds neglecting the management practices mentioned in this study could represent an important source of *Leptospira* infection for other herds in the same geographic area, as well as for other animal species.

## Background

Leptospirosis is considered one of the important zoonoses with a worldwide distribution. It is most prevalent in countries with subtropical or tropical climates since leptospires survive better in warm and wet environments [1-3]. Leptospirosis has been considered an occupational disease; however globalization, climate change and human migration to new areas have placed human populations at risk [4]. The infection has also been established as one of the most important infectious diseases in livestock, particularly in cattle, due to negative impacts on reproduction (abortion, embryonic death, stillbirths and infertility), decreased milk production and growth rates, as well as indirect costs associated with treatments [5-8]. The bacterial agent, *Leptospira* is divided into serogroups, which in turn are divided into serovars based on their antigenic differences [5].

*Leptospira* infection in Chile is present both in domestic and wild animals [9-11] and does therefore constitute a public health risk. However, a mandatory official record of this disease in humans was not established until 2002 together with a diagnostic surveillance system. From that date, the annual incidence rate has been estimated to range from 0.02 to 0.18 per hundred thousand inhabitants per year [12]. Several studies have been conducted to estimate leptospiral seropositivity in some selected animal populations such as cattle (59 to 91%), swine 70%, horses (49%) wild mice (47%), goats (24%) and sheep (7%) [11].

In areas with similar climatic conditions and herd management practices such as Chile, factors such as herd size, replacement policy, herd type, access to contaminated water sources, introduction of other animals, presence of veterinary assistance, and different domestic ruminants co-grazing, are possibly associated with seropositivity against *Leptospira* and herd infection status [7,13-15]. Herd-level factors associated with the risk of a herd being seropositive have shown a significant impact not only on the overall seroprevalence of leptospirosis, but also on the serovar distribution [13].

The south of Chile constitutes the main milk producing area of the country; 80% of the dairy herds producing 66% of the total Chilean milk yield. A significant number of the dairy farms in Chile are classified as subsistence farms, corresponding to 84% of the 11,000 dairy farms in this geographical area [16,17].

Regarding leptospirosis control in Chile, there is neither an official program nor an epidemiological characterization of smallholder dairy farms, and no previous studies have reported *Leptospira* seroprevalence or risk factors for seropositivity or active *Leptospira* infection in these types of herds. This study was carried out to determine *Leptospira* seroprevalence and to evaluate risk factors associated with seropositive results in smallholder bovine dairy herds in southern Chile.

## Methods

### Study population and study design

A cross-sectional study was conducted among smallholder dairy farms in De Los Ríos region, southern Chile, between January and April 2011. These are subsistence farmers that produce <100,000 kg of milk per year [16,17]. The cattle graze outside all-year round and are fed little or no concentrates. Traditionally, milk was collected by hand and transported to a local co-operative milk collection centre where it was added to milk from other farms and cooled in a large refrigerated tank. At the time of the study, it was mandatory for each farm to have its own milking machine and milk bulk tank. The study population comprised more than 2,000 animals from 69 dairy herds. A convenient sample of 1,537 apparently healthy dairy cows was included in the study. In most of the herds, all lactating cows were sampled.

### Serum collection

Individual blood samples (5–10 ml) were obtained, under owners’ consent for the collection of samples, by venipuncture from the coccygeal vein using the Vacutainer system, in strict accordance with the recommendations in the Guide of Use of Animals for Research of Universidad Austral de Chile, approved by the Committee on the Ethics of Animals for Research (http://www.uach.cl/direccion/investigacion/uso_animales.htm). All samples were kept at room temperature until transfer to the Department of Biochemistry and Microbiology, Faculty of Sciences, Universidad Austral de Chile, Valdivia. In the laboratory, blood samples were kept at room temperature until centrifugation and removal of serum. The serum samples were kept at −20°C until analysis.

### Collection of epidemiological data

Information on management and housing factors was collected from the owner or person in charge of each herd using a written questionnaire with open and closed questions, after getting oral consent. The questionnaire was designed to obtain information on variables regarded as potential risk factors for *Leptospira* seropositivity defined by serological titres ≥ 1: 100 as well as for *Leptospira* infection defined by serological titres ≥ 1: 400 [18]. The questions were related to presence of domestic and wild animals sharing pasture with cattle, presence of dogs, history of clinical cases of leptospirosis in the herd, *Leptospira* vaccination (on a yearly basis), purchase and introduction of animals (within the last five years), presence of a specific calving area, cleaning of the calving area, system of calf rearing (natural or sucking method versus artificial or weaning method), adult cattle in contact with calves and heifers, abortion events (during the last five years), contact with neighboring animals, and presence of rodent control.

### *Leptospira* serology

The sera were examined for antibodies against six selected reference *Leptospira* serovars (Table 1) using the Microscopic Agglutination Test (MAT) [18]. The antigens used consisted of serovars known to be present in the study area and to be of clinical importance [10,19].

**Table 1:** ***Leptospira interrogans *and *Leptospira borgpetersenii *serovars used as antigens in the microscopic agglutination test for serological analyses of bovine leptospirosis in a study among dairy cows in Chile 2011**

**Species**	**Serogroup**	**Serovar**	**Strain**
*Leptospira interrogans*	*autumnalis*	Autumnalis	Akiyami A
*Leptospira borgpetersenii*	*ballum*	Ballum	Mus 127
*Leptospira interrogans*	*canicola*	Canicola	Hond Utrecht IV
*Leptospira interrogans*	*icterohaemorrhagiae*	Icterohaemorrhagiae	Icterohaemorrhagiae
*Leptospira interrogans*	*pomona*	Pomona	Pomona
*Leptospira borgpetersenii*	*sejroe*	Hardjo	Ar

All sera that gave a positive reaction at 1:100 screening dilution were titrated in serial twofold dilutions to titre end-point of 1:400. A titre of 1:100–1:200 was considered a low positive titre, and interpreted as indicating exposure to leptospirae. Titres ≥ 1:400 were considered to be high-positive titres, and were interpreted as indicating either recent or active infection [18].

### Statistics

Statistical analyses were performed using SAS version 9.2 (Cary NC, USA). The dependent variable was event/trial, in this case number of seropositive animals per herd/number of sampled animals per herd. All analyses were performed using the glimmix procedure. Two sets of analyses were conducted; one using a titer cut-off of 1:100 and the other 1:400. Univariable analyses were initially performed to investigate the association between *Leptospira* seropositivity and each of the explanatory variables (Table 2). All variables with a p-value ≤0.25 were included in a multivariable regression model. In the analysis using a titer of 1:100 the following variables showed a p-values ≤0.25: use of designated calving area, cleaning designated calving area, calf rearing system, adult cows in contact with calves, adult cows in contact with heifers, horses on the farm, rodent control, sheep and/or goats present on the farm and leptospira vaccination. For the analysis using a cut off of 1:400 the variables cleaning a designated calving area, calf rearing system, horse on the farm and leptospira vaccination showed a p-value ≤0.25. Manual backward elimination of variables with p-values ≥0.05 was performed until all remaining variables showed a p-value of ≤0.05. The models were investigated for interactions and confounders. A confounder was defined as a variable changing the point estimates of the included explanatory variables by more than 25%. The variable herd was included as a random effect to correct for over dispersion.

**Table 2:** **Cross classification of biosecurity or management variables and serological *Leptospira *results using the microscopic agglutination test at herd level using a cut-off level of 1:100 among 69 dairy herds in southern Chile, 2011**

**Variables**	**Category**	**MAT results**	
		**No. seropos (%)**	**No. seroneg (%)**
Abortions	yes	18 (26)	5 (7.2)
	no	34 (49)	12 (17)
Adult cows in contact with calves	yes	27 (39)	10 (14)
	no	25 (36)	7 (10)
Adult cows in contact with heifers	yes	34 (49)	9 (13)
	no	18 (26)	8 (12)
Calve rearing system	natural	25 (36)	11(16)
	artificial	27 (40)	6 (8.7)
Cleaning the designated calving area	yes	5(7.2)	4 (5.8)
	no	47 (68)	13 (8.7)
Horses on the farm	yes	12 (17)	1 (1.5)
	no	40 (58)	16 (23)
Leptospira vaccination	yes	3 (2.1)	0 (0)
	no	49 (33)	17 (25)
Pigs on the farm	yes	15 (22)	4 (5.8)
	no	37 (54)	13 (19)
Presence of dogs	yes	43 (62)	11 (16)
	no	9 (13)	6 (8.7)
Purchase and/or introduction of new cattle	yes	31 (45)	9 (13)
	no	21 (30)	8 (12)
Rodent control	yes	39 (57)	12 (17)
	no	13 (19)	5 (7.2)
Rodents on the farm	yes	39 (57)	7 (10)
	no	13 (18)	10 (15)
Sheep or goats on the farm	yes	11 (16)	5 (7.3)
	no	41 (59)	12 (17)
Use of designated calving area	yes	33 (48)	4 (5.6)
	no	19 (28)	13 (19)
Wild boars	yes	8 (12)	1 (1.4)
	no	44 (64)	16 (23)

## Results

### Descriptive results

The mean number of sampled dairy cattle per herd was 22, with a range from 3 to 84. All the study herds showed the same semi-extensive milking type system, co-habiting with other farm animals. All cattle in the herds were apparently healthy, showing no reproductive or production problems. Only 11% of the herds had a history of suspected clinical leptospirosis presumptively diagnosed by necropsy and there was no history of vaccination against *Leptospira* during the last five years*.* However, 4.3% of the herds reported vaccination more than 5 years ago. In all but one herd, cattle coexisted on pasture together with other domestic and wild animals, such as hare, fox, puma, wild boar, pudu and mink. A high proportion of herds (78%) had dogs and also reported the presence of rodents on their premises (67%). Fifty eight percent of the herd owners defined themselves as having open herds, and they allowed entry of new animals to their farms. More than half (54%) of the herds had a specific calving area, but few of them (13%) routinely cleaned this area. An almost equal number of herds used either a natural or an artificial calf rearing system. An important proportion of herd owners (54%) confirmed that adult cattle had direct contact with calves and heifers (Table 2).

### Serological results

Of the included herds 75% (52/69) showed positive results for one or more *Leptospira* serovars using a cut off value of ≥1:100. Out of those herds 63% (n = 33) had at least one individual with a titer suggesting active infection (≥1:400). Overall, 21% (320/1,537) and 8% (128/1,537) of included dairy cows showed titers of ≥1:100 and ≥ 1:400, respectively, for at least one of the *L. interrogans* and *L. borgpetersenii* serovars (Table 3). Within each herd, the frequency of seropositive dairy cows ranged from 2% to 75%, with a median of 15%.

**Table 3:** **Seroprevalence of *Leptospira *serovars at and different titre levels determined by the microscopic agglutination test among 69 dairy herds in southern Chile, 2011**

**Serovar**	**1:100**	**1:200**	**≥1:400**	**Total**	**Total %**
*Leptospira borgpetersenii* serovar Hardjo	81	61	118	260	81.25
*Leptospira interrogans* serovar Pomona	20	8	7	35	10.94
*Leptospira interrogans* serovar Autumnalis	10	3	2	15	4.7
*Leptospira interrogans* serovar Canicola	2	2	1	5	1.6
*Leptospira interrogans* serovar Icterohaemorrhagiae	2	1	0	3	0.94
*Leptospira interrogans* serovar Ballum	2	0	0	2	0.63
Total	117	75	128	320	100

*Leptospira borgpetersenii* serovar hardjo was the serovar most frequently reported from animals with positive serological results independent of the cut-off value being used (Table 3).

### Risk factors for leptospira seropositivity and infection

The variables considered risk factors in the multivariable model for *Leptospira* seropositivity, using a cut-off value of 1:100 were, using natural calve rearing vs artificial (P = 0.006; Odds Ratio = 3.0) (Table 4) and using a designated calving area (P = 0.05; OR = 1.9). Another factor associated with *Leptospira* seropositivity was vaccination (P = 0.0001; OR = 6.5). Adults cows in contact with calves had no association (P = 0.007; OR = 1.1). No interactions or confounders were found in the model. There were no significant variables in the model associated with titres > =1:400.

**Table 4:** **Significant management and biosecurity variables considered risk factors in a multivariable logistic regression for *Leptospira *seropositivity using a cut off of 1:100 among 69 dairy herds in southern Chile, 2011**

**Variable**	**β**	**p-value**	**OR (95% CI)**
Using natural calve rearing vs. artificial	0.11	0.006	3.0 (1.4-6.3)
Using designated calving area	0.63	0.05	1.9 (0.98-3.5)
Adult cows in contact with calves	−1.1	0.007	0.34 (0.16-0.74)
*Leptospira* vaccination	1.9	0.0001	6.5 (2.2-19)

## Discussion

This is the first risk factor study of leptospiral seropositivity in small holder cattle herds in southern Chile. Despite constituting more than 80% of the milk herds in the country [16,17], the milk production of these herds makes up less than 20% of the milk produced and industrialized in Chile. Given the significant number of small holder cattle herds in the country, this risk factor study was carried out in order to gather more in-depth information on these herds, including details of husbandry practices that possibly favor *Leptospira* transmission both within and between herds.

The high frequency of herds (75%) with at least one MAT-positive animal demonstrates the wide distribution of *Leptospira* infection in the smallholder cattle population in southern Chile. This seroprevalence is higher than the 43% and 53% found by Alonso-Andicoberry et al. [7] and Subharat et al. [15], respectively, in Chilean cattle herds. It is similar, though, to the 82% in unvaccinated suckler herds reported by Ryan et al. [14], where calves were reared with infected dams, just as is the case in the present study.

Although the MAT is a serological diagnostic screening test, it is considered confirmatory if animals have titers ≥ 1:400 [18]. Out of 320 positive samples, 128 (40%) showed antibody titers equal to or greater than 1:400, which suggests an important active infection transmission process in the animal population under study.

The most prevalent *Leptospira* serovar found in this study was *Leptospira borgpetersenii* serovar Hardjo. This is consistent with what has been presented previously in the literature, both nationally and internationally [6,10,19-22]. Although, southern Chile, with its abundant grassland and moderate temperatures, is an ideal environment for *Leptospira* survival [14,23,24] it is suggested that infected cattle act as the main reservoir for *Leptospira borgpetersenii* serovar Hardjo, independently of favourable climatic conditions [7]. However, poor biosecurity practices can lead to increased risk and perpetuation of the infectious agent and thus the onset of infection in the herd. In the present study, this was reflected by the finding that use of natural calf rearing vs. artificial and a designated calving area was shown to be a risk factor for seropositivity.

In this study a high percentage of the animals showed titers above1:400 (40%). This suggests that *Leptospira borgpetersenii* serovar Hardjo is adapted to cattle as high titres are expected after active infections [8].

A large proportion of herd owners (52%) confirmed that they used a natural calf raising system, allowing the lactating cattle to meet their calves (from birth to weaning after 6–8 months). Close contact between an infected and a susceptible animal, as in the natural calf raising system, in the author’s opinion, should be considered as the most important variable influencing the herd’s infection status in southern Chile dairy herds. This is consistent with findings reported elsewhere on transmission of infections in cattle herds, where herd level factors related with close contact such as co-grazing between infected and susceptible hosts, access to contaminated water sources and introduction of other animals showed association with leptospirosis herd infection status [13].

In most cases, the calving area was defined as a small paddock beside the owner’s home. Using this heavily overcrowded calving area increased the likelihood of cattle being seropositive, suggesting high levels of *Leptospira* transmission. A simple interpretation indicates that this variable in itself should not be considered a risk factor as the majority (77%) of the positive herds did not report any practice of cleaning the calving area. The improper hygienic maintenance of the calving area, such as urine shedding of leptospires and accumulation of leptospira contaminated fetal tissue and placentae may lead to an increased risk for infection. This is one plausible explanation for the significant association between calving area and *Leptospira* seropositivity. This is true especially during the rainy season when the humidity is high. All these factors combined create an environment that enhances the survival of these bacteria.

## Conclusions

This study demonstrated that a high proportion of herds in small dairy farms in southern Chile tested *Leptospira* positive. Together with vaccination and antibiotic therapy, specific preventive measures in husbandry practices should be implemented to control *Leptospira* transmission. Herds neglecting the management practices mentioned in this study could represent an important source of *Leptopira* infection for other herds in the same geographic area, as well as for other animal species.

### Ethics

The present study was carried out in strict accordance with the recommendations in the Guide of Use of Animals for Research of Universidad Austral de Chile (http://www.uach.cl/direccion/investigacion/uso_animales.htm).

## Competing interest

The authors declare competing interest neither competing nor financial.

## Authors’ contributions

MS: design of the study, lab work and draft writing; BO design of the study; ES: lab work and draft writing; GR: design of the study; SB: design of the study, data analysis and draft writing. All authors read and approved the final manuscript.
